# Detection of *IS6110* and *HupB gene* sequences of *Mycobacterium tuberculosis and bovis* in the aortic tissue of patients with Takayasu’s arteritis

**DOI:** 10.1186/1471-2334-12-194

**Published:** 2012-08-20

**Authors:** María Elena Soto, Ma Del Carmen Ávila-Casado, Claudia Huesca-Gómez, Gilberto Vargas Alarcon, Vicente Castrejon, Virgilia Soto, Sergio Hernandez, Nilda Espinola-Zavaleta, Maite Vallejo, Pedro A Reyes, Ricardo Gamboa

**Affiliations:** 1 Department of Physiology, Departments: Physiology, Immunology and Molecular Biology, National Institute of Cardiology “Ignacio Chavez”, Juan Badiano No. 1, Colonia Sección XVI, 14080, México DF, Mexico

**Keywords:** Takayasu^′^s arteritis, Tuberculosis, *IS6110* and *HupB* gene, Extrapulmonary

## Abstract

**Background:**

Takayasu’s arteritis (TA) is a chronic inflammatory disease affecting the large arteries and their branches; its etiology is still unknown. In individuals suffering from TA, arterial inflammation progresses to stenosis and/or occlusion, leading to organ damage and affecting survival. Relation of TA with *Mycobacterium tuberculosis* has been known, but there have been only a few systematic studies focusing on this association. The *IS6110* sequence identifies the *Mycobacterium tuberculosis* complex and the *HupB* establishes the differences between *M. tuberculosis* and *M. bovis*. Our objective was to search the presence of *IS6110* and *HupB* genes in aorta of patients with TA.

**Methods:**

We analyzed aorta tissues embedded in paraffin from 5760 autopsies obtained from our institution, we divided the selected samples as cases and controls; Cases: aortic tissues of individuals with Takayasu’s arteritis. Control positive: aortic tissues (with tuberculosis disease confirmed) and control negative with other disease aortic (atherosclerosis).

**Results:**

Of 181 selected aorta tissues, 119 fulfilled the corresponding criteria for TA, TB or atherosclerosis. Thus 33 corresponded to TA, 33 to tuberculosis (TB) and 53 to atherosclerosis. The mean age was 22 ± 13, 41 ± 19, and 57 ± 10, respectively. *IS6110 and HupB* sequences were detected in 70% of TA tissues, 82% in tuberculosis, and in 32% with atherosclerosis. Important statistical differences between groups with TA, tuberculosis versus atherosclerosis (p = 0.004 and 0.0001, respectively) were found.

**Conclusion:**

We identified a higher frequency of *IS6110* and *HupB* genes in aortic tissues of TA patients. This data suggests that arterial damage could occur due to previous infection *with M. tuberculosis*.

## Background

Takayasu’s arteritis (TA) is a rare primary vasculitis with a chronic inflammatory course [1], in most cases clinical diagnosis is established in advanced stages of the disease [2,3]. It is more frequent in women younger than 40 years [4,5].

TA pathology is found in the vessel wall at the adventitia, inflammatory changes start in the vasa vasorum (vasa vasoritis) and the inflammation progresses from the adventitia to the intimae characterized by mononuclear infiltration and poorly defined granulomas which dominate in the middle layer. The intimae (the endothelium) remain normal for a long time, until it is altered by atherosclerosis [6].

It has been postulated that the progressive inflammation in the aorta of TA patients can be due to a chronic infectious process [7].

Tissues, in their interaction with pathogens, produce responses mediated by immune mechanisms with or without infectious disease development. Occasionally, after an infectious disease has been resolved, there are pathological conditions linked to the immune response by diverse mechanisms, among these the ones which stand out are molecular mimicry and diseases induced by equivalent mechanisms when there is tissue damage and probable release of “neoantigens” [8,9].

For more than five decades, a possible association of TA with tuberculosis-mediated infection has been proposed, because tissue injuries in both diseases show similarities. Cutaneous lesions similar to the nodose erythema or Bazin’s erythema induratum have been found in TA [10].

On the other hand, in patients with pulmonary tuberculosis there can be arteritis in the vessels near the cavitations, such as the Rasmussen aneurysm [11], tuberculosis, pseudo aneurysms, aortic and vascular damage secondary to abdominal tuberculosis similar to that found in TA, which have been discovered during surgical procedures and usually worsen prognosis [12,13].

Tuberculosis (TB) is an infection caused by a bacteria (from the *Mycobacterium tuberculosis*, >*bovis > africanum* complex). Fourteen different species have been reported to infect humans, but only *M. bovis* and *M. tuberculosis* have been completely related with humans; the presence of the other species could be related with a specific phenotype of the disease [14].

Only in a fraction, 5 to 10%, of the infected persons develop the tuberculosis disease, in the rest of the cases the infection is latent, and only, under immune deficiency conditions, the disease is expressed, with the pulmonary site being the most common. Extrapulmonary type involvement can occur through the hematogenic pathway and this infection is frequently found in lymphatic ganglia, serosa, pleura, peritoneum, brain, kidney, bone, and skin [15-17].

The behavior of this *Mycobacterium* is still unknown, but its evasion strategies inside the host are well known, which allows the pathogen to reach other tissues with varying clinical expressions [18]. The DNA of *M. tuberculosis* persists in normal pulmonary tissue during latent infection; therefore it has been proposed to search for its DNA in tissues, where its presence could indicate a latent state, leading to the search of new elimination strategies [19].

Analysis of the *IS6110* sequence was described for the first time in *M. tuberculosis* in 1990 [20,21]. This sequence is also present in the genome of other members of the *M. tuberculosis* complex [22], suggesting that the *IS6110* insertion element is an ancestral precursor of the microorganism; hence, the technique allows for gene differentiation [23,24]. Previous assays to differentiate the C terminal region of the *HupB* gene in *M. tuberculosis (Rv2986c) and M. bovis (Mb3010c*) have yielded good results [25] and the usefulness of the test has been confirmed [26].

Morbidity and mortality due to TA occurs in young people at their productive stage. Although there is histological evidence suggesting an association with tuberculosis, the results remain controversial; therefore, improved research strategies are needed to define the possible involvement of infection in the arterial damage.

Our objective was to identify and compare specific sequences of *M. tuberculosis* and *M. bovis* in aortic tissues embedded in paraffin obtained from autopsies of patients with TA, tuberculosis, and atherosclerosis.

## Methods

### Selection of cases and controls

We reviewed the autopsy files of the Pathology Department in search of cases of autopsy with TA, and controls with TB (pulmonary and extra pulmonary) reported positive with culture of secretions or tissues, for the given case, and cases with diagnosis of atherosclerosis.

After localizing the files of each disease group these were verified with the clinical files searching for clinical and laboratory data that would support without doubts the diagnoses of each group as established in the autopsies to define the phenotype of the TA cases and of the control groups.

The arterial lesion was classified according to Hata et al. who suggested five types of vessel damage involved: type I (aortic arch branches), type IIa (ascending aorta, aortic arch and his branches), type IIb (ascending aorta, aortic arch and his branches, thoracic descendent aorta), type III (thoracic descendent aorta, abdominal aorta and/or renal arteries), type IV (abdominal aorta and/or renal arteries), and type V (combine characteristics from type IIb and IV) and if there is coronary or pulmonary lesion the letter C or P is added [27] (Table 1).

**Table 1:** Criteria for Takayasu^′^s arteritis accord to American College of Rheumatology

**Criteria**	**Definition**
Age at disease onset in year	Development of symptoms or findings related to Takayasu’s arteritis at age <40 years.
Claudication of extremities	Development and worsening of fatigue and discomfort in muscles of one or more extremity while in use, especially the upper extremities.
Decreased brachial artery pulse	Decreased pulsation of one or both brachial arteries.
BP difference >10 mmHg	Difference of >10 mmHg in systolic blood pressure between arms.
Bruit over subclavian arteries or aorta	Bruit audible on auscultation over one or both subclavian arteries or abdominal aorta.
Arteriogram abnormality	Arteriographic narrowing or occlusion of the entire aorta, its primary branches, or large arteries in the proximal upper or lower extremities, not due to arteriosclerosis, fibro-muscular dysplasia, or similar causes: changes usually focal or segmental.

From the clinical files, data on classification criteria, gender, age, contact or exposure to patients with tuberculosis, origin from an endemic zone (site known in Mexico as prevalent for tuberculosis), symptomatology, evolution, time of the illness, and cause of death were extracted.

### Identification of cases and controls

TA cases, those with more than four ACR criteria, controls with tuberculosis, tissue cultures and confirmed bacilloscopy. Atherosclerosis control with, histopathology-confirmed findings and the presence of atheroma in the intima layer type I, II and III according to American Heart Association.

We searched for those autopsy files with well described macroscopic and microscopic findings. Once the cases and controls were identified, we searched for the aortic tissue blocks and analyzed their storage conditions; those selected were sectioned with a microtome and to prevent any possible contamination with the microtome blade, this was aseptically cleaned with octane and 100% alcohol before and after cutting each sample.

**Figure 1:**
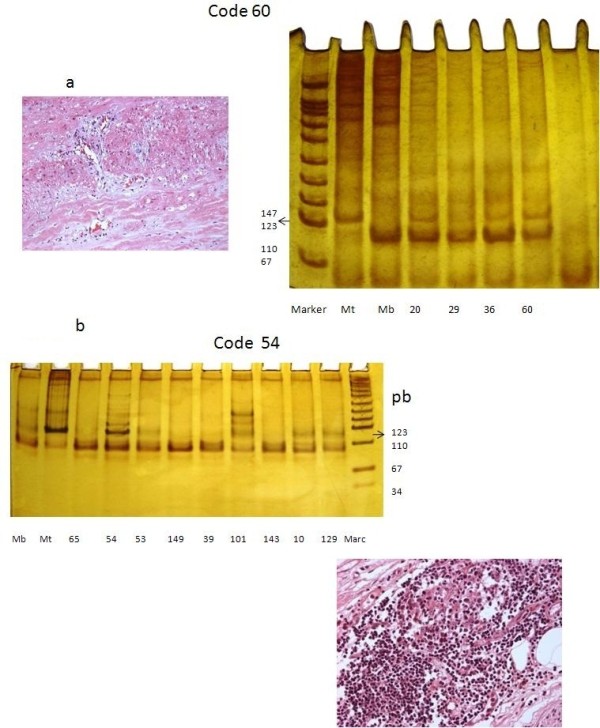
**There two cases one with Takayasu’s arteritis and the other one with tuberculosis confirmed by culture.** The nitrate silver stained amplification product of *M. tuberculosis* was electrophoreses on no denaturing 10% polyacrylamide gel. The 123 product obtained for *M. tuberculosis* is indicated by the arrows. **a**) Lanes: molecular size marker 123 bp for *M. tuberculosis* (MT) and 89 for *M. bovis* and; 1: DNA of bacillus of *Mycobacterium* in the aorta of case’s code number 60; DNA H37Rv taken from cellular culture. Case of a woman of 12 years old with Takayasu’s arteritis type I + C + P and low socioeconomic Level, with initial symptoms of palpitations. We found generalized aortic damage, located in the adventitia and she died to the 16 years; **b**) Case of a man of 18 years old, he had familiar with tuberculosis. He came to the Institute by dyspnea and he developed cardiogenic shock. He has extensive myocardium infarct and died.

Samples were blinded with a code, before being processed and analyzed by molecular biology experts, proceeding then with the deparaffinizing, and DNA extraction and amplification Figure 1.

The aortic lesion observed in the autopsy for the three groups is shown in the Table 2.

**Table 2:** Aortic lesion observed in the autopsy (macroscopic findings)

	**Takayasu's**	**Tuberculosis**	**Atherosclerosis**
		**n (%)**	
Without lesion	0 (0)	8 (24)	2 (3)
Ascending aorta	5 (15)	0 (0)	1 (1)
Descending aorta	0 (0)	2 (6)	4 (7)
Abdominal aorta	7 (21)	11 (33)	27 (50)
All	21 (64)*	12 (36)**	19 (35)***
Total	33	33	53

* vs** P= 0.04; OR= 3.06, CI 95% (1.00-9.52). * vs *** P= 0.02; OR= 3.13, CI 95% (1.16-8.59).

### DNA extraction from the paraffin embedded tissues

The DNA was obtained with a commercial kit (Illustra Nucleon Genomic, GE Healthcare). Briefly, 20 to 30 μm of paraffin embedded tissue, contained in a 1.5-ml assay tube, was covered with xylene to deparaffinize the tissue and incubated for 2 min, centrifuged, and xylene was removed. The tissue was rehydrated with successive ethanol (100%, 75%, 50%, and 25%) washings. Afterwards, proteinase K was added and the DNA was extracted with the Nucleon resin. The obtained DNA was stored at -70°C, until used.

#### DNA amplification

To amplify the *IS6110* sequence (123 bp) *of M. tuberculosis,* the primers IS6110 F (5’- CCTGCG AGC GTA GGC GTC GG-3’) and R (5^′^-CTC GTC CAG CGC CGC TTC GG-3’) were used [28]. Briefly, for a 25 μl reaction, 10.7 μl of double distilled H_2_0, 2.5 μl of buffer 10X, 1.5 μl of 25 mM MgCl_2_, 300 μM of each of the four deoxyribonucleotides, 0.125 μl of Taq polymerase (Invitrogen), and 400 μg DNA were added. The amplification cycle used was: 3 min at 95°C, 30 cycles each at 94°C-30 sec (denaturation), 63°C-30 sec (annealing), and 72°C for 1 min (extension), and finally one cycle at 72°C for 10 min.

The difference of 27-pb in the C terminal region of the *hupB* gene in *M. tuberculosis* (Rv2986c) and *M. bovis* (Mb3010c) was analyzed by nested PCR (N-PCR). In this case, the used primers *were*: N (5’-GGAGGGTTGGGATGAACAAAGCAG-3’) and S (5’-GTATCCGTGTGTCTTGACCTATTTG-3’) [29]. For the N-PCR assay for the C terminal region of gene *HupB*, we used the following primers: F (5’-CCAAGAAGGCGA CAAAGG-3’) and R (5’-GACAGCTTTCTTGGCGGG-3’). In this case, 5 μl of the PCR amplified product with primers N and S was used as DNA template for the nested PCR. Each reaction mixture (25 μl) for the N-PCR contained 1.25 mM MgCl_2_, 200 μM deoxynucleotide triphosphate, 0.5 μM of primers F and R, 10 mM Tris–HCl (pH 8.8), 50 mM KCl, 0.08% Nonidet P-40 and 1.0 U of *Taq DNA polymerase*. The reaction mixture was subjected to an initial denaturation at 94°C for 10 min and 35 cycles, each of 1 min at 94°C and an annealing and extension at 59°C and 72°C for 1 min, followed by an extension at 72°C for 10 min. Products were analyzed in a 10% polyacrylamide gel and stained with ethidium bromide and/or silver nitrate.

The expected sizes of the *HupB* sequences for *M. tuberculosis* and *M. bovis* were 116 and 89 bp, respectively. We included a double control to distinguish from mixed *M. tuberculosis* and *M. bovis* infection*.* In all the PCR we used a negative control, that is, the same reaction mixture except DNA, which was replaced by sterile water. Also, we used a positive control for the analysis a sample of Mycobacterium (*M. tuberculosis* H37Rv) obtained from cell lines extracted from strains of *M. bovis* AN5 and *M. tuberculosis HRv37*, kindly provided by the Department of Cellular Biology of the National Institute of Cardiology “Ignacio Chavez” (Figure 1).

To evaluate the precision and fidelity of the test, we reviewed the results at two different time point’s moments by the same molecular biologist and also evaluated the results through intra-observer and between inter-observer.

### Histological analysis

Tissues were stained with hematoxylin-eosin, Schiff’s periodic acid, and Masson stain, and Auramine Rhodamina. The tissues were assessed by a certified pathologist, who assessed the presence of fibrosis and inflammatory infiltrates.

### Statistical analysis

We analyzed the size of the sample by calculating proportions, in which an alpha error of 0.01 and a power of 0.90 as well as a prevalence of extrapulmonary disease of 25% were considered, yielding a number of 33 per group.

An exploratory analysis of the data was performed. We estimated the proportions by categories for the qualitative variables and the central tendency measures and dispersion for the quantitative variables. A verification of the normality in the distribution of the latter variables by means of the Shapiro Wilk’s test (*p* > 0.05) was made. Comparison among qualitative variables (gender, type of work, socioeconomic level, endemic zone, age at the time of diagnosis, previous or coincident tuberculosis disease in each group, type of tuberculosis, type of TA according to the Numano classification, antecedent of familial tuberculosis and cause of death) was performed by Chi-square test or Fisher’s exact test, as needed. Comparison of quantitative variables (age at diagnosis and at time of death, time of disease evolution) among groups was performed by means of one-way variance analysis with the Bonferroni post hoc test.

Canonical correlation (CC) was used for the multivariate analysis, it allows analyzing two sets of variables, those of response or effect, which in this study were: type of tuberculosis, presence of the *IS6110* and *HupB* sequences associated to mycobacteria, aortic lesion, age at the time of diagnosis, and presence of granulomas, these were: U (p) (X_1_, *X*_2_, …. X_p_), and the other set corresponds to those variables that explain or are predictive, such as socioeconomic level, body mass index, exposure to tuberculosis, and time of evolution: V (q) (Y_1_, Y_2_ ….Y_q_), the same as in multiple regression models, except that the canonical correlation can have more than two variables on both sides of the equation. The main objective of CC is to identify the lineal combinations within the response:

$$\begin{array}{c} U_{1}=a_{11}X_{1}+a_{12}X_{2}+\ldots \ldots +a_{1p}X_{p}\\ U_{2}=a_{21}X_{1}+a_{22}X_{2}+\ldots \ldots +a_{2p}X_{p}\\ U_{r}=a_{r1}X_{1}+a_{r2}X_{2}+\ldots \ldots +a_{\text{rp}}X_{p} \end{array}$$

and with the predictive variable:

$$\begin{array}{c} V_{1}=b_{11}Y_{1}+b_{12}Y_{2}+\ldots \ldots +b_{1p}Y_{p}\\ V_{2}=b_{21}Y_{1}+b_{22}Y_{2}+\ldots \ldots +b_{2p}Y_{p}\\ V_{r}=b_{r1}Y_{1}+b_{r2}Y_{2}+\ldots \ldots +b_{\text{rp}}Y_{p} \end{array}$$

The largest correlation will be identified between U_1_ and V_1_, the second largest between U_2_ and V_2_, as long as there is no correlation between U_1_ and U_2_, neither between V_1_ and V_2_; and the third largest correlation will be given between U_3_ and V_3_, and, as aforementioned, there can be no correlation between U_3_ and U_1_ and U_2_, neither between V3 and V_1_ and V_2_. These combinations are known as canonical variables.

#### Ethical approval

The study protocol was in compliance with the Declaration of Helsinki, approved by the ethic committee of Instituto Nacional de Cardiología “Ignacio Chavez” and registered (ClinicalTrials.gov ID: 53248).

##### Informed consent

Informed consent was given by our Institution through Ethical Committee because patient’s samples were obtained by autopsy. In this case, we presented the letter before to begin the study.

## Results

From a total of 5760 autopsies, 181 cases that complied with the inclusion criteria were selected; of these, two were eliminated because the samples had not been preserved adequately; in 20 not enough tissue was obtained to extract the DNA, in 24 no information on the clinical and laboratory studies was available, 10 more presented multiple co comorbilities, and 6 presented tuberculosis disease diagnosed before their admittance at the hospital. The final sample was of 119 tissues, 33 corresponded to Takayasu’s arteritis according to the ACR criteria, 33 corresponded to tuberculosis, and 53 to atherosclerosis.

Ages of TA, TB, and atherosclerosis at the time of diagnoses were found with a median of 20 (range of 4-46), 41 (range of 8-70) and 56 (range of 40-90) respectively, the difference had statistical significance with (p = 0.001); the socioeconomic level was similar between TA and TB patients, whereas the atherosclerosis group tended to correspond to a medium to high socioeconomic level, but without statistical differences among groups. Table 3 depicts the demographic variables and the occupation of the patients.

**Table 3:** Clinical characteristics

**Variable**	Takayasu’s arteritis* (TA) n = 33	**Tuberculosis control (TB) n = 33**	Atherosclerosis control** (A) n = 53	**p**
A. Clinical Characteristics				
Age at diagnosis (years)	22 ± 13	41 ± 19	57 ± 10	0.0001*
Time of disease evolution (months) range	36 (2–468)	60 (1–336)	36 (2–288)	NS
Age at the time of death	29 ± 14	49 ± 18	62 ± 10	0.0001*
Body mass index at the time of death	22 ± 6	24 ± 5	27 ± 4	0.0001**
Systemic arterial hypertension n (%)	26 (79)	19 (58)	51 (96)	0.02*
B. Tuberculosis Disease				
With tuberculosis disease well known previously n (%)	0	33 (100)	0	0.0001*
Without tuberculosis lesions n (%)	13 (39)	-----	43 (81)	0.0002*
Exposed at tuberculosis n (%)	7 (22)	33 (100)	6 (12)	0.0001*
Native from and residents of tuberculosis- endemic zone n (%)	17 (52)	19 (57)	37 (69)	NS

* TA vs Tuberculosis or Atherosclerosis. ** Atherosclerosis vs TA.

### Classification of the arterial lesion in the TA tissues

Classification of the TA cases revealed type V arterial lesion in 25 (76%), six of them had no coronary involvement; four were of type V + C; eleven of type V + C + P; and two corresponded to V + P according to Hata classification [27]. Table 4 presents distribution of types I, II, and IV, time of disease evolution, and the cause of death associated to the arterial lesion and the average age at death.

**Table 4:** Type of TA, characteristics of disease evolution and causes of death

**Type of Takayasu’s (Numano)**	**n (%)**	**Age at start of the disease**	**Time of evolution in months**	**Causes of death**
V + C + P	11 (33)	44	12	Stroke
		30	24	CCF, hemoptysis, hypovolemic shock
		15	24	Aneurysm of the LV, CCF, hemoptysis
		6	2	AMI
		18	96	Pulmonary infarct embolic; Stroke
		10	468	TCRF, AMI
		46	12	Multiple foci broncho-pneumonia
		32	36	Hypertensive cardiopathy, brain
		38	36	hemorrhage
		9	24	AMI, transmural
		9	12	Carotid aneurysm rupture, hypovolemic shock
				Acute pulmonary edema, LVH
V	7(21)	24	132	Aortic regurgitation, cardio-respiratory failure
		46	36	CCF, pulmonary tuberculosis,TB meningitis
				DCM Acute pulmonary edema
		40	12	Double aortic lesion + SLE TCRF
		16	12	Uremic syndrome, Acute pulmonary edema
		20	216	CRTF, DCM Acute pulmonary edema
		37	24	
		7	12	
V + C	4 (12)	13	24	CCF, TCRF, cardiac cachexia
		12	12	CCF, Hypertensive crisis, encephalopathy.
		8	60	TCRF, hypertensive crisis
		17	24	Double aortic lesion, rupture of the membranous septum
V + P	3 (9)	10	96	CCF, Acute pulmonary edema
		22	108	CCF, PTE multiple
		18	132	PTE, PAH.
IV + C	2 (6)	4	30	AMI, Cardiac failure
		23	120	TCRF, uremic syndrome
IV + C + P	2 (6)	29	36	Tricuspid failure, pericarditis
		36	120	Aortic failure
IIa + C + P	2(6)	12	48	Myocarditis and aortic coarctation
		45	120	Tearing of the aorta, hypovolemic shock
I + C	1 (3)	28	17	AMI,PTE, cardiogenic shock
IIb + C + P	1 (3)	30	2	AMI

First row indicate the type el lesion accord to Hata et al. + Coronary (C) or Pulmonary (P). Abbreviators in causes of death: CCF (Congestive cardiac failure), LV (Left ventricle), AMI (Acute myocardial infarction), TCRF (Terminal chronic renal failure), LVH (Left ventricular hypertrophy), TB (Tuberculosis), DCM (Dilated cardiomyopathy), SLE (Systemic lupus erithematosus), PTE (Pulmonary thromboembolism), PAH (Pulmonary arterial hypertension).

In this series, the main causes of death in TA was acute myocardial infarction in 8 (24%) and chronic renal insufficiency in 8 (24%) and of these cases, active disease was found in 75% and 87%, respectively and it was demonstrated by inflammatory infiltration in the microscopic analysis.

We found six TA cases, whose clinical records indicated that they had suffered from hemoptysis, and who until their death had no diagnosis of TB. The first case was a woman with hemoptysis, who’s X-ray revealed a Ghon focus, and tuberculosis pericarditis was reported in the autopsy, the aorta had granulomatous injuries and inflammatory infiltrates in the adventitia. In the clinical interview she had informed of close contact with a relative having active tuberculosis. The second case corresponded to a 9-year-old boy who arrived at the emergency service with hypertensive encephalopathy and died. Stenosis of the left renal artery was described in the autopsy, and pulmonary tuberculosis lymphadenitis was found. The third case was a woman who had presented dysphonia, dyspnea, productive cough, and hemoptysis, angina pectoris, palpitations, and systemic arterial hypertension. The initial study revealed an aortic dissection, for which she was operated upon, but the ascending aorta was ruptured and she died due to massive bleeding. The fourth case corresponded to a patient complaining of hemoptysis and fever, the chest X-ray revealed a pulmonary nodule. He died due to a pneumonia complication acquired in his home town. For the other two cases with hemoptysis, autopsy did not report any tuberculoses lesion.

Three cases, in which pulmonary granuloma or Ghon focus was found, showed positive sequences of the mycobacterium gene in the aortic tissue; one of them, a 10 year old boy died from acute pulmonary edema. The autopsy also reported tuberculosis peri-pancreatic ganglia. The second case corresponded to a 12-year-old girl, who reached the hospital with congestive cardiac failure, anasarca, and was diagnosed with myocarditis. The third case died from bronchopneumonia acquired in his home town and presented hemoptysis, this case corresponds to one of the six aforementioned cases.

Each case report was classified and maintained with a code until the final analysis in which results were confronted with those of the gene study and histopathologic results.

### Classification of controls with tuberculosis

Characteristics of the tuberculosis disease in these controls are shown on Table 5.

**Table 5:** Characteristics of patients with tuberculosis

**Number of patient**	**Age at diagnosis**	**Time of evolution in months**	**Site of tuberculosis**	**Cause of death**
1	41	144	miliary	Ventricular fibrillation (+)
2	32	60	Lung kidney	TCRF (+)
3	58	120	lung	Cardiogenic shock
4	50	36	pericardium	Broncho-pneumonía
5	21	36	lung	CCF, PTE pericarditis
6	59	60	miliary	Thyroid storm
7	70	96	ganglion	PTE, massive
8	51	3	Tuberculous meningitis	Septicemia, VF
9	18	1	lung	Cardiogenic shock, AMI
10	28	336	lung	TCRF
11	23	288	lung	PAH
12	42	12	lung	Endocarditis
13	40	144	lung, kidney	AoR, severe; AMI
14	58	24	lung	Acute pulmonary edema
15	49	2	lung	VF, AMI
16	11	156	lung	Meningitis brain edema
17	10	60	lung, ganglion	Brain infarct, malignant SAH
18	26	36	lung	PTE
19	40	192	lung, suprarenal glands	AMI
20	20	300	lung, ruptured aneurysm	Hypovolemic shock
21	8	120	lung	TCRF, malignant; SAH
22	33	288	miliary, tamponade	Tamponade
23	53	252	lung	Cardio-respiratory failure
24	48	24	miliary	Multiple organ failure
25	59	96	lung	AMI anterolateral
26	30	12	lung	Ventricular fibrillation
27	69	3	lung, abdominal aorta	TCRF, brocho-pneumonia
28	22	132	lung	Acute pulmonary edema
29	26	144	lung, meningitis	Enclaving of the amygdala
30	52	12	lung	Tamponade
31	55	144	lung	ASCRV, re-infarctation
32	79	4	lung	Ruptured abdominal aneurysm
33	62	3	lung, kidney	TCRF, brain hemorrhage

TCRF (Terminal chronic renal failure), PAH (Pulmonary arterial hypertension) AoR (Aortic regurgitation) CCF (Congestive cardiac failure), PTE (Pulmonary thromboembolism), VF (Ventricular failure), AMI (Acute myocardial infarction), SAH (Systemic arterial hypertension), ASCRV (After surgery of cardiac revascularization).

### Classification of controls with atherosclerosis

In these atherosclerosis control patients, 51 (96%) had arterial hypertension and were overweight, 93% coursed with dyslipidemia, 54% were active smokers; all had a sedentary life style. Also, the atherosclerosis patients had aneurismal disease, carotid disease and aortoiliac disease. The atherosclerosis was confirmed at the moment of the autopsy by the pathologist.

### Tissues

Time (in years) of storage of tissues was: for TA = 30 ± 11, for TB = 25 ± 11, and for atherosclerosis = 23 ± 9. The type of aortic tissue obtained during autopsy was from the abdominal aorta; the macroscopic damage at the time of autopsy was mainly described in the abdomen and in the whole aorta. The adventitia was affected most frequently (71%) in TA cases; controls with atherosclerosis showed an increase in atheromas in the intimae layer (86%) which is a common result in this disease.

Analysis of the autopsy tissues from the TA cases revealed macroscopic tuberculous injuries in the aorta and distant places such as lungs, peripancreatic site, kidney, complex Ghon, mediastinum, meningitis tuberculoses, pericardium and also tearing of aorta, which had not been identified previously when the patient was alive (Table 6).

**Table 6:** Tuberculoses lesion reported in distant places at the aorta

**Site of location of Tuberculous injuries**	**Takayasu’s n (%)**	**Tuberculosis n (%)**	**Atherosclerosis n (%)**
Lung	6 (18)	20 (60)	5 (9)
Lung and peri pancreatic site	2 (6)	0	0
Lung and kidney	2 (6)	3 (9)	1 (2)
Lung ruptured aneurysm	1 (3)	1 (3)	0
Lung suprarenal glands	0	1 (3)	0
Lung and abdominal aorta	0	1 (3)	0
Complex Ghon	3 (9)	0	2 (4)
Mediastinum	2 (6)	0	0
Tuberculous meningitis	1 (3)	2 (6)	0
Pericardium	2 (6)	1 (3)	0
Tearing of aorta and hemoptysis	2 (6)	0	0
Neck	0	0	1 (2)
Kidney	0	0	1 (2)
Miliary	0	3 (9)	0
total	22 (61)	32 (97)	10 (19)

The inflammatory infiltrate in aortic tissue in TA cases was observed in 73% vs. 33% of the controls with tuberculosis (*p* = 0.003) and 27% in the atherosclerosis controls (*p* = 0.000).

### Detection of genes *IS6110* and *HupB*

The inter-observer correlation in the detection of the *IS6110* sequence had a Kappa value of 94%; whereas, for the *HupB* sequence this correlation was of 80%. Intra-observer concordance was of 90%.

*IS6110* and *HupB* sequences that identify *M. tuberculosis* were detected in 82% of the tissues from tuberculosis patients and in 70% in those from TA patients; there were no statistically significant differences between these two groups, however they showed significant differences when compared to tissues of patients with atherosclerosis in which 32% were positive (p = 0.004). The characteristic sequence that identifies *M. bovis* was present in 45% of the tissues from tuberculosis patients. The presence of this sequence was similar in patient with tuberculosis, TA and atherosclerosis (Table 7).

**Table 7:** Frequency of the gene sequences associated to *Mycobacterium *in the aorta tissue

**Positive tests for the sequences of*IS6110*and nested PCR for*M. tuberculosis***				
	Takayasu’s	Tuberculosis	Atherosclerosis	p
	n = 33	n = 33	n = 53	
	n (%)	n (%)	n (%)	
*IS6110 + HupB* (Tb) positive samples	23 (70)	27 (82)	17 (32)	0.0001*
*HupB (Bovis)* positive samples	12 (36)	15 (45)	18 (34)	NS

Add: * Takayasu’s vs Atherosclerosis.P= 0.001; OR= 4.87; CI 95% (1.73-14.0). * Tuberculosis vs Atherosclerosis. P= 0.0001; OR = 9.5; CI 95% (3.0-31.0).

On the other hand, in the TA subjects, during autopsy, tuberculous lesions adjacent to the aorta or in other sites were found in 16 subjects (48%). In twelve (75%) of these, positive sequences for *IS6110* (suggests the presence of *M. tuberculosis*) were found, of these, in turn, eight were of pulmonary location, 3 were extrapulmonary, and one was both pulmonary and extrapulmonary.

In the group with tuberculosis, we identified 17 (51%) with pulmonary tuberculosis, 6 (18%) with extrapulmonary, 6 (18%) with both types, pulmonary and extrapulmonary, and 4 (12) with milliary. Both sequences (*IS6110* and *HupB*) were identified in 13 samples with pulmonary tuberculosis (13/17 = 76%), in the 6 samples with extrapulmonary (6/6 = 100%), in 5 with pulmonary and extrapulmonary (5/6 = 83%) and in 3 with milliary (3/4 = 75%).

In the group with atherosclerosis, we found 10 (19%) tuberculous lesions distant from the aorta, 5 (9%) were in the lung, two of them were positive to both *M. tuberculosis* sequences, the other sites were negative.

The proportion of cases and controls originated from a tuberculosis-endemic zone was: 17 (52%) in TA, 19(57%) in the control group with tuberculosis and 37(69%) in the atherosclerosis group.

Of the 23 TA patients with *IS6110* + *HupB* positive sequences, we found that 18 (78%) corresponded to cases with a low socio-economic status

A bivariate analysis was performed to analyze the predisposing factors in relation to the presence or not of the sequences. The variables that resulted with statistical significance we included in a multivariate analysis model of canonical correlation.

Canonical correlation revealed a significantly high correlation between the first (0.8704) and second (0.6894) pair of canonical variables. Likewise, we identified lineal coefficients with statistical significance in the effect and explicative variables in both pairs of canonical variables. For the first pair, variables of type of tuberculosis, presence of the sequences of both genes, site of the aortic lesion, and presence of granuloma showed an inverse association with the socioeconomic level, the BMI, and the age stratum, and in the same sense with the exposure variable. For the second pair, only three variables kept coefficients with statistical significance, i.e., type of tuberculosis, presence of sequences of both genes, and site of the aortic lesion, the first two associated positively with exposure and age stratum, and the third variable showed an inverse association with these same variables. The canonical correlation reached 87% and the Wilk’s Lambda test with an F = 0.0000; the Lawley Hotteling trace and Loy's largest root yielded the same statistical significance (Table 8).

**Table 8:** Canonical correlation analysis by cases and controls with sequences *of *genes *IS6110 *and *HupB *genes

**Variables**	**Coefficient**	**P value**	**95% CI**	**Can Corr**	**λ**
Type of tuberculosis	-0.155	0.006	-0.264; -0.045	0.8704	0.0000
*gen IS6110+ HupB*	-0.485	0.000	-0.705; -0.265		
Location of aortic injury	-0.192	0.000	-0.285; -0.098		
Group of disease	0.037	0.000	0.031; 0.043		
Presence of granuloma	-0.311	0.025	-0.581; -0.041		
Socio-economic level	0.188	0.011	0.044; 0.332		
Exposure	-0.333	0.004	-0.556; -0.110		
Body mass index	0.054	0.000	0.032; 0.075		
Age stratum	0.903	0.000	0.762; 1.044		
Time of evolution of disease	-0.001	0.021	-0.003; -0.0002		
Type of tuberculosis	0.565	0.000	0.361; 0.769	0.6894	
*gen IS6110+ HupB*	0.472	0.024	0.063; 0.881		
Location of the aortic injury	-0.482	0.000	-0.656; -0.308		
Group of disease	0.011	NS	-0.0002;0.0214		
Presence of granuloma	0.485	NS	-0.017; 0.987		
Socio-economic level	-0.178	NS	-0.445, 0.089		
Exposure	1.976	0.000	1.562; 2.390		
Body mass index	-0.015	NS	-0.055; 0.025		
Age stratum	0.525	0.000	0.263; 0.786		
Time of evolution of disease	-0.0004	NS	-0.003;0.002		

## Discussion

In 1963, the TA cases presented by Nasu et al. were discussed, arguing that the histopathological findings were insufficient to warrant a possible tuberculous etiology [30]; however, this relation of tuberculosis with arteritis has been observed worldwide [31-34]. In Mexico, since 1971, it has been thought that it might just be a coincidence [35]. Tuberculosis lesions located in distant regions from the damaged artery have been described by Rose [36], coinciding with our findings.

A high percentage of inflammatory infiltration was found in TA tissues as described before by Kerr [37] who found that 40% of cases with TA considered by the Physician without inflammatory activity on the clinical parameters and laboratory findings, the Pathologist found data of activity characterized by inflammatory infiltration, it is related probably with subclinical inflammatory activity. Our findings were higher than Kerr’s findings with about 87%. The etiology of these findings is unknown however; our hypothesis is that could be due to increased autoimmune response, after previous stimulation with *M. tuberculosis*, or latent infection, with reactivation by immunosuppression.

We found tuberculosis lesions near and distant from the aortic tissue, which correlated with the findings of mycobacteria gene sequences.

Likewise, Sharma described autopsy cases in 1998, detecting TA in four patients and emphasizing that there was silent activity of the disease [38]. After this report, case series have been published in which the relation is not clear and scarcely convincing, reinforcing the notion of coincidence [39-42]. In 1971, Sánchez-Torres reviewed 29 TA cases, and searched for a relation of the arteriopathies with tuberculosis; he described that 30% came from a rural zone and more than 50% from a low socioeconomic level. Clinical findings revealed that these patients had suffered from dermopathies and suppurative lymphadenopathy and tuberculosis was confirmed in two patients, while the Mantoux test had been positive in 18 cases [32]. In another study, this author searched for a relation of Bazin’s induratum erythema and nodose erythema with arteritis, from which he concluded that it was possible that the etiology of the dermal injuries were due to hypersensitivity to the tuberculous bacillus [10].

In a case report, Shimizu and Sano found a history compatible with tuberculosis, tuberculoid changes in the aorta or its branches have been found in isolated cases [43-46]. Other studies have reported convincing data of tuberculosis in up to 20% of TA cases [47], whereas in a more recent report scarce convincing evidence was found [39], whereas another report indicates that they occur simultaneously [42].

The widely proposed hypothesis that there is an infectious pathogen associated with the arterial damage, which can condition adjacent immune mechanisms prolonging the inflammatory response, independently from whether the pathogen persists or not in the affected tissue, has been controversial and could be due to the diverse methodological approaches.

The possibility of vasculitis in the aorta tissue of TA could be an extrapulmonary manifestation of tuberculosis, because it is well known that strategies of Mycobacterium tuberculosis evasion within the host, allowing invasion of different tissues results in great variability in the clinical expression of the tuberculosis disease [18,48].

Only a fraction of the infected cases (5-10%) develop the tuberculosis disease, in the majority of them there is a latent infection for all their life and only in immune deficiency condition the disease could be expressed in different localizations -extra pulmonary and pulmonary, although the last one is more known [49,50].

Other authors proposed that the genetic factor in the host could determine the activity and expression of the disease [51,52], and also it was demonstrated that the mycobacterium has capacity to adapt in different host tissues during the progression, latency and reactivation of the disease [53]. This adaptation is related with the identification of 13 factors sigma within *M. tuberculosis and M. bovis* genome which three of them were pointed to have capacity of response to ambient changes that probably occur during the adaptation of bacillus in the tissue the host where it has stayed [54].

Also it is known that in pulmonary tuberculosis there are vessel lesions located near the cavitations described as Rasmussen aneurysm [55], which is a form of arteritis in the wall of the cavitation that on numerous occasions causes hemoptysis. At the moment there are not many follow-up studies in patients with tuberculosis, in order to determine if they develop arteritris.

The prevalence of infections with *M. tuberculosis* in Mexico is unknown. However, according to official statistics in Mexico, in 2005, the pulmonary TB mortality rate was 3/100 000 inhabitants and the morbidity rate was 16.6/10 0000. However these numbers could vary depending of geographical zone, economic and cultural aspects in Mexico.

Currently, few systematic studies to resolve this controversy are underway, although the new diagnostic methods based on molecular tests to identify mycobacteria, used successfully since 1994, when Salo et al. identified *M. tuberculosis* in a preColumbian Peruvian mummy [56] and others groups [57] could be instrumental in resolving these controversies. The technique for the molecular study to identify the sequences of the *M. tuberculosis* complex in paraffin embedded tissues has been already used in other series, aimed at searching for variable length polymorphisms (RFLP^′^s) of the *IS6110* insertion sequence, which were described in *M. tuberculosis* for the first time in 1990 [21] and whose concordance degree with the PCR-RFLP technique has been greater than 90% [26].

The previous reports support our findings. Although we found 18% with negative sequence for IS6110, a possible explains is that not all TB patients had extra pulmonary tuberculosis and the samples analyzed were taken from aortic tissues. If the *Mycobacterium tuberculosis* found a stable site to develop it would not be required to move to other tissues.

In this study, we maintained blinded study protocol for the involved investigators, to diminish the inherent bias in the selection and to reach certainty and maintain internal validity of the results. The tissues were carefully evaluated before starting the study and they complied with the recommended requirements [58-60].

Finally, a multivariate analysis, which allows for the linear evaluation of the variables was included in this study, which confirmed the presence of *IS6110* and *HupB sequences* associated to predictive factors already known for tuberculosis disease, as are exposure [61], low socio-economic level [62], and age, which, in this study, were strongly associated to the presence of the studied gene sequences.

The analysis of cases and controls coming from endemic zones of Mexico revealed a high percentage of positive sequences for both genes, and we found statistically significant differences among them, predominating in the groups with known tuberculosis disease. This allowed us to consider that it could be a predictive variable for tuberculosis disease. We do not have an explanation for the presence of these sequences of genes associated with *M. tuberculosis* in the aorta and the development of arteritis, but a probable hypothesis could be that the pathogen is able to evade the immune response [63] and that the aorta represents a highly oxygenated tissue allowing the bacillus to adapt to the stress generated in the host’s tissue [64].

However, for the explanation regarding as to why arteritis could be an expression of a tuberculosis disease different from the known one, the answer could be that other factors are involved, such as the pathogen’s virulence [65], susceptibility of the host [66], the anatomical site where it lodges in the aorta. To this regard, in the aorta there occurs diversity in thickness, and perfusion and also the cell composition is not the same along the aorta, which could be correlated with the diversity of the disease [67].

We identified the sequences of the genes associated with *M. tuberculosis* in tissues of the aorta from patients with Takayasu’s arteritis. These results are significant and the lesions of the arterial disease of the patients could be a clinical expression of extra-pulmonary tuberculosis. Until now, the presence of a latent infection in TA is possible and cannot be discarded.

Regarding limitations of this study, it is known that bias can come up when performing retrospective analyses and searches; we lacked information on some variables of interest in both cases and controls, for example, if they have BCG vaccination and Mantoux test because these datas were not included in the files.

### Conclusions

This study shows the presence of gene sequences associated with *M. tuberculosis* within the aortic tissue of TA patients and supports the possibility that arteritis results from a latent infection.

The results allow proposing new study hypotheses with respect to the pathogenesis of TA and could have important implications in the development of antituberculosis agents for therapeutic management, which would help in combating any latent infection by *M. tuberculosis*.

## Abbreviations

TA: Takayasu’s arteritis; TB: Tuberculosis; ICA: Invasive coronary angiography; CAI: Coronary artery involvement; ECG: Electrocardiogram; LMC: Left main coronary; PCI: Percutaneous coronary intervention; RCA: Right coronary artery; LAD: Left anterior descending; Cx: Circumflex.

## Competing interests

The authors declare that they do have no competing interests.

## Authors’ contributions

MES Strategy desing, drafted the manuscript, obtained the tissue collection and the financial support, performed the statistical analysis. MDCAC and VS: Search, selection and analysis of tissues. VC and CH-G: Obtained the Mycobacterium DNA and molecular genes analysis. MV: Performed the statistical analysis. GV and PAR: Participated in the study design and helped draft the manuscript. NE-Z. Reviewed the English text and drafted manuscript. SHBCR.Technical assistant in the tissues and histological elaboration. RG: Performed the molecular analysis, participated in the strategy design and drafted manuscript. All authors read and approved the final manuscript.

## Pre-publication history

The pre-publication history for this paper can be accessed here:

http://www.biomedcentral.com/1471-2334/12/194/prepub
